# Myriad Manifestations of 3-Beta-Hydroxysteroid Dehydrogenase 2 Deficiency—A Tale of Two Infants

**DOI:** 10.7759/cureus.21779

**Published:** 2022-01-31

**Authors:** Deep Hathi, Soumik Goswami, Nilanjan Sengupta, Sourya Acharya, Sunil Kumar, Dhruv Talwar

**Affiliations:** 1 Endocrinology, Nil Ratan Sircar Medical College, Kolkata, IND; 2 Medicine, Jawaharlal Nehru Medical College, Datta Meghe Institute of Medical Sciences (Deemed to be University), Wardha, IND

**Keywords:** congenital adrenal hyperplasia, adrenal glands, endocrinology, case series, 3β hsd2 deficiency

## Abstract

3-Beta-hydroxysteroid dehydrogenase type 2 (3β-HSD2) deficiency is a rare variety of congenital adrenal hyperplasia. Based on the severity of the enzymatic defect, it can present with a salt-wasting crisis in both sexes to undervirilization in males and virilization in females. We report two cases of infants with extremes of presentation of this congenital adrenal hyperplasia. First was a 28-day-old child presenting with a salt-wasting crisis while the other was a one-month-old child presenting with ambiguous genitalia. Clinical exome sequencing of the first child confirmed the diagnosis and we report a novel mutation of this gene, while the second child was diagnosed biochemically by raised synacthen-stimulated 17-OH-pregnenolone. The first case was managed with glucocorticoid and mineralocorticoid supplementation, while the second child was managed conservatively. Due to variable presentations, 3β-HSD2 deficiency should be kept as a differential diagnosis while evaluating a child with congenital adrenal hyperplasia.

## Introduction

Congenital adrenal hyperplasia (CAH) refers to a spectrum of disorders characterized by the deficiency of enzymes involved in the synthesis of cortisol in adrenal glands, which might lead to a life-threatening adrenal crisis [1].

21-hydroxylase deficiency remains the most common cause of CAH followed by 11 β hydroxylase deficiency. 3-Beta-hydroxysteroid dehydrogenase type 2 (3β-HSD2) deficiency is a very rare cause of CAH caused due to defects in the HSD3β2 gene accounting for <0.5% cases with an overall prevalence of <1/1,000,000 births [2-5].

The clinical spectrum varies according to the severity of enzyme defects ranging from a salt-wasting crisis (SW) in both sexes to undervirilization in males and virilization in females. The uncommon, more severe form of this disease results from complete enzymatic activity loss leading to SW as a result of reduced mineralocorticoids. The milder form may result from the loss of 3β-HSD2 function partially resulting in the virilization of infants who are genetically female while it results in the undervirilization of infants who are genetically male. Therefore, this is the only form of primary CAH, which may lead to presentation in the form of ambiguous genitalia in both sexes. The single best marker of 3β-HSD2 deficiency is elevated ∆5-17-hydroxypregnenolone. However, molecular genetic testing is confirmatory for diagnosis [6].

To improve our understanding of the clinical spectrum and diagnosis of this rare disease, we will summarize the clinical presentation, genetics, and treatment of two children with 3β-HSD2 deficiency.

## Case presentation

Case 1 summary

A 28-day-old child, first child of non-consanguineous marriage with the sex assigned as male, was brought by his mother with complaints of poor weight gain, poor suckling, vomiting, and generalized hyperpigmentation from day 8 of life. There was no history of maternal exposure to androgen intake. There was no history of ambiguous genitalia in the family. 

On admission, the child was alert, irritable, severely dehydrated, and weighed 2.5 kg (birth weight 2.8 kg). Examination revealed a pulse rate of 156/ min and blood pressure (BP) of 54/34 mm Hg.

**Figure 1:**
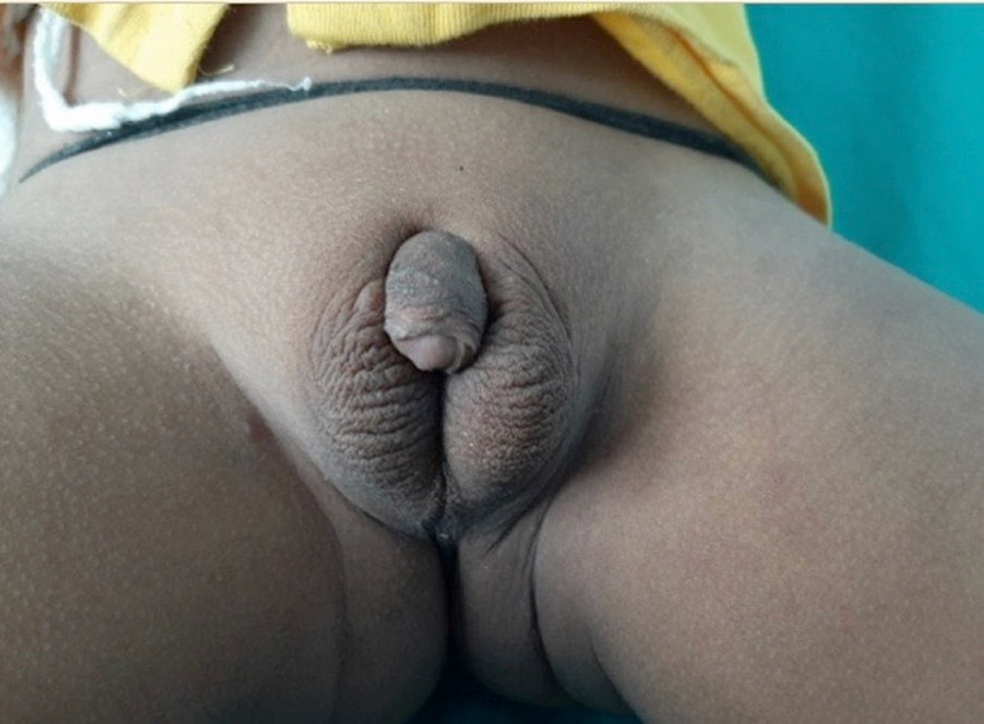
Shows external genitalia of the first child with unfused scrotal folds, micropenis, bilateral palpable testes in scrotum and penoscrotal hypospadias (EMS 3/12) EMS, external masculinization score

Hyperpigmentation was present at genitalia, digits, and oral mucosa. Genital examination showed an external masculinization score (EMS) of 3/12 (bilaterally descended testes to scrotal sac) (Figure 1). There was no obvious bony deformity on examination. The child was administered intensive fluid replacement, parenteral hydrocortisone 50 mg TDS along with correction of electrolyte abnormalities, while blood was drawn for hormonal assays. After three days of therapy, serum electrolytes were normalized and the child became hemodynamically stable.

Laboratory investigations suggested (Table 1) a provisional diagnosis of 3β-HSD2 deficiency. Ultrasonography revealed absence of Mullerian structures. Clinical exome sequencing confirmed a homozygous missense variation in exon 4 of the HSD3β2 gene (c.371G>T) that resulted in substitution of isoleucine to serine at codon 124. This variant is a novel one and has not been reported till date in the literature.

**Table 1:** Laboratory investigations of Case 1 and Case 2 ACTH, adrenocorticotropic hormone; FSH, follicle-stimulating hormone; LH, luteinizing hormone; DHEAS, dehydroepiandrosterone-sulfate; LCMS, liquid chromatography-mass spectrometry

Lab investigation	Case 1	Case 2	Reference range
Sodium (meq/l)	117	135	135-145
Potassium (meq/l)	7.2	5.5	3.5-5
Random blood sugar (mg/dl)	40	78	>50
Cortisol (8 am) (ug/dl)	3.1	8.8	5-25
Plasma ACTH (pg/ml)	240	86.5	7.2-63.6
Testosterone (ng/ml)	0.2	0.4	2.27-10.3
FSH (Miu/ml)	13.8	25.6	
LH (Miu/ml)	7.4	15	
DHEAS (ug/dl)	650	518	133-440
Aldosterone (ng/dl)	-	12.1	<23.6
Renin (µIU/ml)	-	10.63	2.8-39.9
17-OH-progesterone (ng/ml)	27.4	16.5	
ACTH-stimulated 17-OH-pregnenolone (nmol/l) (LCMS assay)	-	446	<94
Karyotype	46,XY	46,XY	

The baby was started on fludrocortisone 0.1 mg/day and hydrocortisone 5 mg/day along with extra salt in feeds. At three-month follow-up, the child was asymptomatic and had adequate weight gain (6.5 kg).

Case 2 summary

A one-month-old child, second product of consanguineous marriage with male sex assigned at birth, presented with ambiguous genitalia. There was no episode of SW and maternal exposure to external androgen intake. There was no history of ambiguous genitalia in family.

**Figure 2:**
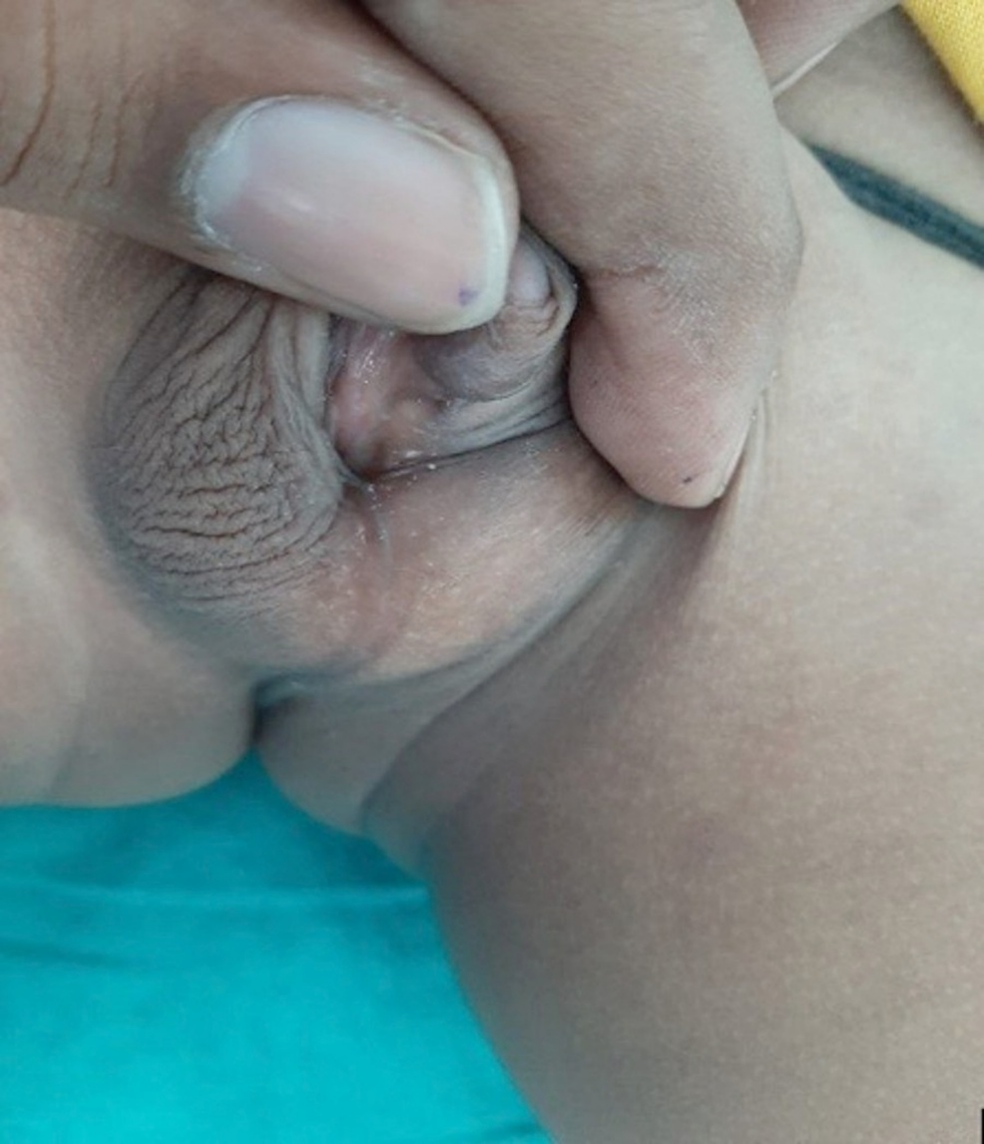
Shows external genitalia of the second child with fused scrotal folds, bilateral palpable testes in scrotum, micropenis, and penoscrotal hypospadias (EMS 6/12) EMS, external masculinization score

On admission, the child was alert, playful, and weighed 3.8 kg. On examination, pulse was 120/min and BP was 94/64 mm Hg. There was no hyperpigmentation. Systemic examination was unremarkable. EMS was 6/12 (bilateral palpable testes in the scrotal sac) (Figure 2).

Laboratory profile has been summarized in Table 1. The child was administered 500 IU of beta human chorionic gonadotropin for three consecutive days and samples were collected on the fourth day for testosterone, androstenedione, and dihydrotestosterone from which the stimulated ratio was calculated, which were T/DHT 3.46 and T/A 1.7, thus ruling out the biosynthetic defect. Considering the raised 17-OH-pregnenolone and dehydroepiandrosterone-sulfate values, the patient was planned for adrenocorticotropic hormone (ACTH)-stimulated 17-OH-pregnenolone.

Post-stimulated 17-OH-pregnenolone was 446 nmol/l by liquid chromatography-mass spectrometry, which was diagnostic for nonclassical 3β-HSD2 deficiency [6]. In view of adequate cortisol response to ACTH and normal BP, the child was not started on any hormonal replacement therapy. However, the parents were explained regarding the stress dosing of hydrocortisone.

The parents of both children were counseled regarding the need for corrective surgery for external genitalia and referred appropriately. 

## Discussion

3β-HSD deficiency is a very rare cause of CAH. Till date, 110 cases have been confirmed by genetic testing and more than 50 types of 3β-HSD2 gene mutations have been detected, which includes frameshift, missense, nonsense, splicing pathogenic variants, and deletions. Frameshift, nonsense, and in-frame deletions are associated with a severe, SW phenotype. The less severe non-SW and nonclassical phenotypes are associated with missense mutations of HSD3β2 gene, with residual but diminished enzymatic activity [7].

Our case series includes two male children with extreme presentations-one presenting with SW in the neonatal period, while the other presenting with ambiguous genitalia without SW. After reviewing the OMIM, ClinVar, and NCBI databases, the mutation site c3714>T found in our patient was found to be a novel one [7].

Therapy for 3β-HSD2 deficiency includes hormonal replacement and genital reconstructive surgery. Hydrocortisone and fludrocortisone are given at a replacement dose of 10-15 mg/m^2^/day and 100 mcg/day, respectively [8].

In 46,XY fetus, androgens are required for sex differentiation before 12 weeks of gestation. 3β-HSD2 deficiency is associated with varying degrees of undervirilized male genitalia necessitating masculinizing treatment [9]. Extra-adrenal conversion of excess dehydroepiandrosterone to testosterone in 46,XY karyotype with 3β-HSD2 deficiency by 3β-HSD1 produces some amount of testosterone that contributes to incomplete virilization [10].

Our series depicts the extreme clinical and biochemical spectrum of 3β-HSD2 deficiency and reminds us of keeping it in mind when dealing with an undervirilized male.

## Conclusions

3β-HSD2 deficiency is a very rare form of CAH with variable clinical and biochemical presentation ranging from an SW crisis in severe enzyme defect to isolated genital ambiguity in its milder form. Our series dealt with two cases demonstrating these extremes. Hence, the treating clinicians should be well aware of such varied presentations of 3β-HSD2 deficiency to ensure timely diagnosis and management with hormone replacement when indicated thereby preventing potential mortality in the infancy. 3β-HSD2 deficiency is, therefore, an infrequent but important differential diagnosis to consider for CAH.
